# A hierarchical multimodal transformer fusion network for cyberbullying detection across social media platforms

**DOI:** 10.3389/frai.2026.1926150

**Published:** 2026-10-29

**Authors:** Aziza Zhidebayeva, Aigerim Toktarova, Rustam Abdrakhmanov, Kamalbek Berkimbayev, Marzhan Bekbolat, Abdimukhan Tolep

**Affiliations:** 1 Department of Computer Science and Mathematics, Faculty of Natural Science and Engineering, Peoples’ Friendship University named after Academician A. Kuatbekov, Shymkent, Kazakhstan; 2 Department of Information and Communication Technologies, M. Auezov South Kazakhstan University, Shymkent, Kazakhstan; 3 International University of Tourism and Hospitality, Turkistan, Kazakhstan; 4 Department of Computer Engineering, Khoja Akhmet Yassawi International Kazakh-Turkish University, Turkistan, Kazakhstan

**Keywords:** cyberbullying, graph neural networks, multimodal learning, nlp, social media, transformers

## Abstract

Cyberbullying has become a critical challenge for online social media platforms due to its harmful psychological and social consequences and the increasing volume of user-generated content. Existing cyberbullying detection methods predominantly rely on textual information and often fail to effectively capture conversational context, user interaction structures, visual content, and behavioral metadata, resulting in limited robustness and interpretability. This paper proposes HMTF-Net, a Hierarchical Multimodal Transformer Fusion Network for explainable cyberbullying detection across social media platforms. The proposed framework jointly learns complementary representations from textual content, conversation threads, heterogeneous user interaction graphs, images, and metadata through dedicated representation learning branches. An adaptive cross-modal attention mechanism with gated fusion integrates heterogeneous modalities, while a hierarchical conversation encoder captures discourse-level dependencies and a graph attention encoder models structural relationships among interacting users. Furthermore, an explainability module provides interpretable predictions by highlighting influential words, conversation turns, graph nodes, and image regions, whereas a domain-adversarial learning strategy enhances cross-platform generalization by learning platform-invariant representations. Experimental evaluations on publicly available cyberbullying datasets demonstrate that HMTF-Net consistently outperforms representative state-of-the-art approaches, achieving 97.6% accuracy, 97.0% precision, 96.2% recall, 96.6% *F*1-score, and a 0.986 ROC-AUC while maintaining competitive computational complexity and inference efficiency. Ablation studies confirm the contribution of each proposed component, and robustness experiments demonstrate strong generalization across multiple social media platforms and noisy linguistic conditions. These results indicate that HMTF-Net provides an accurate, interpretable, and scalable solution for trustworthy automated cyberbullying detection in real-world online environments.

## 1 Introduction

Online social media platforms have fundamentally transformed the way individuals communicate, exchange opinions, and participate in digital communities. Social media platforms and other discussion forums facilitate large-scale information sharing but have simultaneously increased the prevalence of cyberbullying, hate speech, harassment, and abusive interactions. Cyberbullying has become a major societal concern because of its detrimental effects on victims’ mental health, emotional well-being, and social participation, particularly among adolescents and young adults (Philipo et al., 2026a,b). The enormous volume of user-generated content makes manual moderation impractical, motivating the development of automated cyberbullying detection systems based on artificial intelligence and natural language processing. Recent advances in deep learning have substantially improved content understanding and classification performance by learning semantic representations directly from raw data (Badawi, 2024; Iwendi et al., 2023). Nevertheless, accurately recognizing cyberbullying remains a challenging task because offensive behavior is often implicit, sarcastic, context-dependent, or distributed across multiple posts and communication modalities rather than appearing explicitly within a single message.

Existing cyberbullying detection methods have predominantly focused on textual analysis using conventional machine learning models or transformer-based language models. Although these approaches have demonstrated promising performance, they often ignore valuable contextual information embedded in conversation history, user interaction patterns, visual content, and behavioral metadata (Omarov et al., 2024). In real-world social media environments, understanding whether a message constitutes cyberbullying frequently requires considering previous replies, relationships among users, attached memes or images, and temporal posting behavior. Moreover, coordinated harassment campaigns involve complex interaction networks that cannot be effectively modeled through isolated text representations alone (Zaib et al., 2022). Recent multimodal approaches have attempted to integrate textual and visual information; however, many still lack effective hierarchical conversation modeling, heterogeneous graph representation learning, and robust feature fusion strategies (Pendela et al., 2024). Another important limitation is the lack of explainability, making it difficult for human moderators to interpret model decisions, while performance often deteriorates when models are transferred across different social media platforms because of domain distribution shifts (Aggarwal et al., 2024).

To overcome these limitations, this paper proposes HMTF-Net, a Hierarchical Multimodal Transformer Fusion Network for explainable cyberbullying detection across social media platforms. The proposed framework jointly learns complementary representations from textual content, conversation threads, heterogeneous user interaction graphs, visual information, and metadata through dedicated representation learning branches. A cross-modal attention mechanism combined with adaptive gated fusion enables effective interaction among heterogeneous modalities while reducing the influence of noisy features. Furthermore, hierarchical conversation encoding captures discourse-level contextual dependencies, whereas heterogeneous graph attention learning models structural relationships among users participating in online discussions. To enhance transparency, an explainability module identifies influential words, conversation turns, graph nodes, and visual regions contributing to the final prediction. In addition, a domain-adversarial learning strategy improves cross-platform generalization by learning platform-invariant representations (Ghafoor et al., 2025). Comprehensive experiments conducted on publicly available multimodal cyberbullying datasets demonstrate that the proposed framework consistently outperforms representative state-of-the-art methods in terms of classification performance, robustness, explainability, and cross-platform adaptability, making it a promising solution for trustworthy automated content moderation.

## 2 Related works

Automatic cyberbullying detection has evolved from traditional machine learning techniques to advanced deep learning frameworks capable of learning complex semantic representations from online user-generated content. Early studies primarily relied on handcrafted linguistic features, including n-grams, term frequency-inverse document frequency (TF-IDF), sentiment lexicons, and syntactic information combined with conventional classifiers such as Support Vector Machines, Random Forests, and Naïve Bayes (El Koshiry et al., 2024; Mohiuddin et al., 2025; Philipo et al., 2026a,b; Almomani et al., 2024). Although these approaches demonstrated reasonable performance on benchmark datasets, they were highly dependent on feature engineering and often struggled to generalize across different social media platforms. The emergence of deep neural networks significantly improved cyberbullying detection by automatically learning semantic features through convolutional neural networks (CNNs), recurrent neural networks (RNNs), long short-term memory (LSTM), and bidirectional LSTM architectures (Sultan et al., 2023a,b; Murshed et al., 2023; Gutiérrez-Batista et al., 2024). More recently, transformer-based language models such as BERT and RoBERTa have achieved state-of-the-art performance by capturing contextual dependencies within textual content (Joshi et al., 2026; Anand et al., 2023). Nevertheless, these text-centric approaches frequently fail to capture implicit bullying expressed through conversational context, user behavior, or multimodal content.

Recognizing the limitations of text-only analysis, recent research has increasingly explored multimodal cyberbullying detection by integrating textual, visual, and metadata information. Social media posts often combine text with images, memes, emojis, videos, and user profile attributes, all of which contribute to the interpretation of abusive behavior. Consequently, multimodal learning frameworks have demonstrated superior performance compared with unimodal methods by jointly exploiting complementary information across different modalities (Ram et al., 2026; Omarov et al., 2020; Sujud et al., 2024). Cross-modal attention mechanisms and multimodal transformers further improve feature interaction by learning correlations between textual semantics and visual representations (Pericherla and Ilavarasan, 2025; Obaida et al., 2024). However, most existing multimodal methods focus primarily on text-image fusion while overlooking conversation history, temporal user behavior, and complex interaction patterns among social media users. In addition, many studies evaluate their models on a single platform, limiting their ability to generalize to heterogeneous online environments where language usage and interaction characteristics differ substantially.

Another active research direction focuses on incorporating contextual and structural information into cyberbullying detection. Hierarchical conversation modeling has been proposed to capture discourse-level dependencies across discussion threads, allowing models to distinguish isolated offensive expressions from sustained harassment (Bello et al., 2025; Al-Wesabi et al., 2024). Simultaneously, graph neural networks have demonstrated remarkable success in modeling social relationships by representing users, comments, replies, and interactions as graph structures (Daraghmi et al., 2024; Almufareh et al., 2025). Graph Attention Networks (GATs) and heterogeneous graph neural networks effectively aggregate neighborhood information while assigning adaptive importance to different relationships, enabling improved detection of coordinated abusive behavior and community-level harassment. Despite these advances, relatively few studies have jointly integrate hierarchical conversation encoding with heterogeneous graph learning and multimodal feature fusion within a unified architecture. Moreover, the interpretability of deep learning models remains limited, restricting their adoption in real-world content moderation systems where transparent decision-making is essential for human moderators and platform administrators.

Recent studies have also emphasized explainable artificial intelligence and domain adaptation to improve the trustworthiness and robustness of cyberbullying detection systems. Explainability techniques such as attention visualization, SHAP, Integrated Gradients, and gradient-based attribution methods have been employed to identify influential words, sentences, and visual regions contributing to model predictions (Jaradat et al., 2025, Sundararajan et al., 2017; Durgekar et al., 2026). Meanwhile, domain-adversarial learning has emerged as an effective strategy for mitigating performance degradation caused by domain shifts across different social media platforms by learning platform-invariant feature representations (Aliyeva and Yağanoğlu, 2025; Murshed et al., 2022). Despite these developments, there remains a lack of comprehensive frameworks that simultaneously integrate multimodal representation learning, hierarchical conversation modeling, heterogeneous graph attention, explainable prediction, and cross-platform domain generalization. Motivated by these research gaps, the proposed HMTF-Net introduces a unified multimodal architecture that combines these complementary techniques to achieve robust, interpretable, and generalizable cyberbullying detection across diverse online social media platforms.

## 3 Materials and methods

The proposed Hierarchical Multimodal Transformer Fusion Network (HMTF-Net) is designed to automatically detect cyberbullying by jointly learning complementary representations from textual content, conversation threads, heterogeneous user interaction graphs, visual information, and metadata collected from online social media platforms. Unlike conventional text-based classifiers, the proposed framework integrates multiple heterogeneous information sources within a unified deep neural architecture to capture semantic, contextual, structural, behavioral, and visual characteristics of abusive online interactions. The complete architecture of the proposed framework is illustrated in Figure 1, where multimodal inputs are first preprocessed and encoded independently before being integrated through a cross-modal attention fusion mechanism. The fused representation is subsequently utilized for multi-task prediction while simultaneously learning platform-invariant representations through adversarial domain generalization.

**Figure 1:**
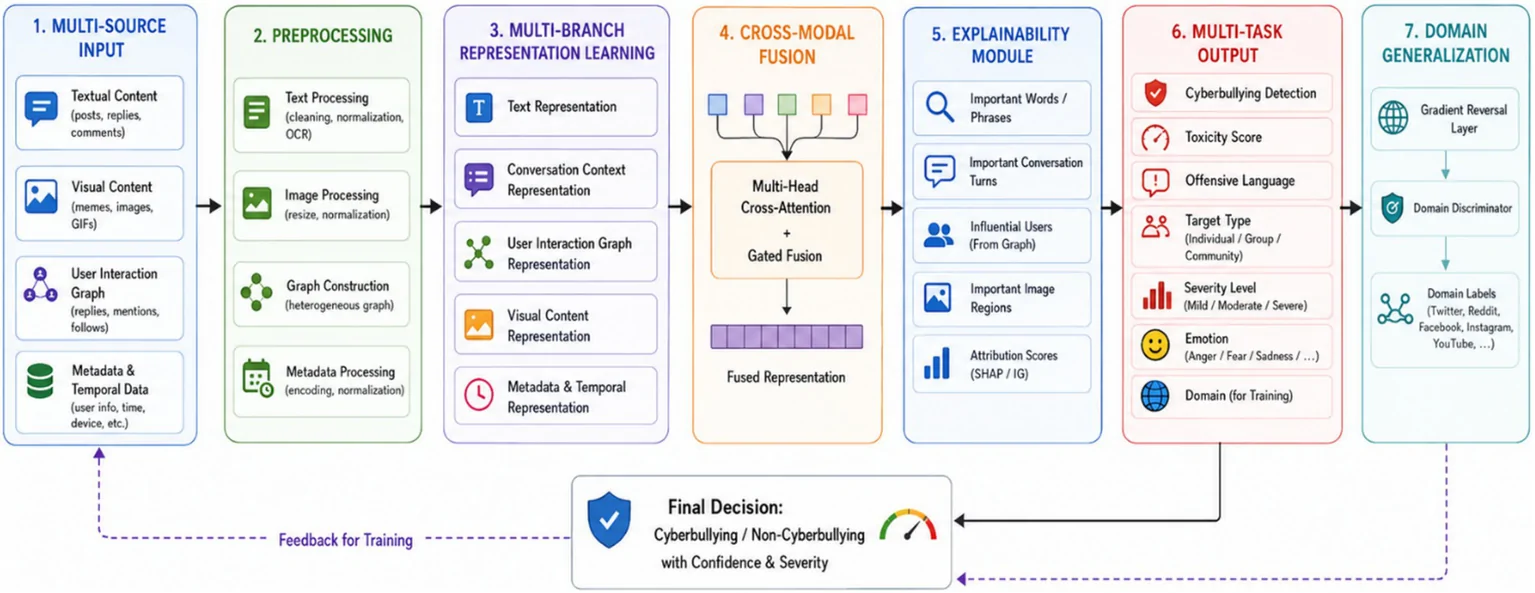
Overall framework of the proposed cyberbullying detection system.

The proposed framework operates on multimodal social media samples consisting of textual posts, conversation histories, attached images or memes, user interaction graphs, and metadata. Publicly available Multibully dataset were employed to evaluate the proposed model (Figure 2). Each sample contains multiple modalities, including post text, chronological conversation threads, visual content, interaction relationships among users, and temporal metadata such as posting time, engagement statistics, and user profile attributes.

**Figure 2:**
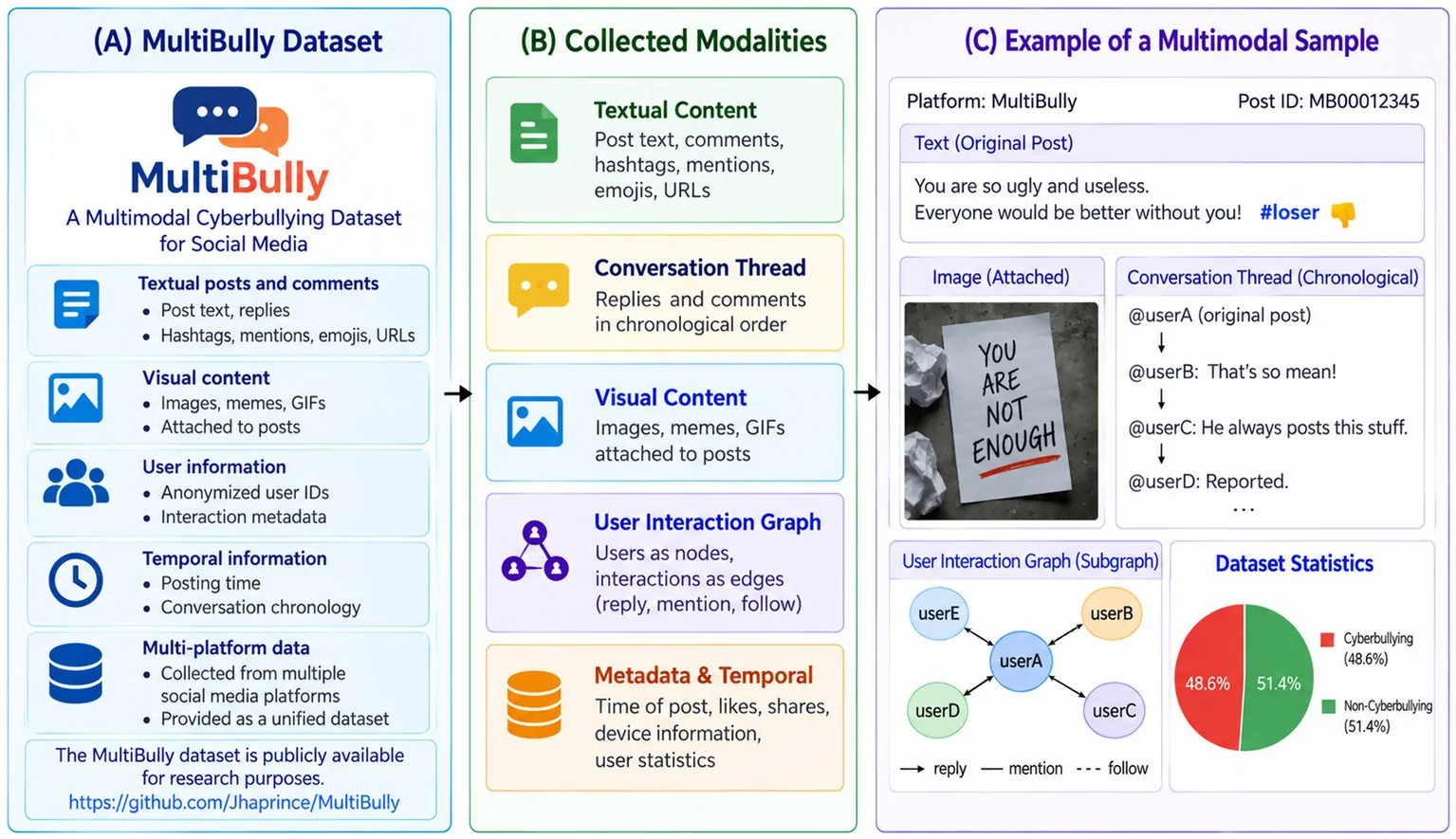
Multimodal cyberbullying dataset: **(A)** MultiBully dataset, **(B)** collected modalities, and **(C)** example of a multimodal sample.

Every instance is annotated using multiple labels including cyberbullying, toxicity, offensive language, target category, severity level, and emotion. Before feature extraction, textual data are normalized by converting characters to lowercase, removing redundant symbols, correcting malformed Unicode characters, and tokenizing sentences. Image inputs are resized to a fixed spatial resolution and normalized according to ImageNet statistics. User interaction graphs are constructed by representing users, replies, mentions, and follow relationships as heterogeneous nodes and edges. Metadata attributes are normalized using min-max scaling,


$$x_{i}^{\prime }=\frac{x_{i}-x_{\min}}{x_{\max}-x_{\min}}$$

Where $x_{i}$ denotes the original numerical attribute and $x_{i}^{\prime }$ represents its normalized value.

After preprocessing, the proposed framework independently learns feature representations from five complementary modalities. Let.


$$X=\{X_{T},X_{C},X_{G},X_{V},X_{M}\}$$

Represent textual content, conversation context, graph information, visual content, and metadata, respectively. Each modality is transformed into a latent representation.


$$Z_{i}=f_{i}(X_{i}),\;\;i\in \{T,C,G,V,M\}$$

Where $f_{i}(\cdot )$ denotes the corresponding representation learning branch.

The textual representation branch extracts semantic information from the original post using an embedding layer followed by one-dimensional convolutional layers and bidirectional gated recurrent units. Given an input token sequence.


$$S=(w_{1},w_{2},\ldots ,w_{n})$$

Each token is first mapped into an embedding vector,


$$E=[e_{1},e_{2},\ldots ,e_{n}]$$

Where $e_{i}\in {\mathbb{R}}^{d}$ . Local semantic patterns are extracted through convolution,


$$h_{i}=\sigma (W_{c}*E+b_{c})$$

Where $W_{c}$ represents convolution kernels, $b_{c}$ denotes the bias term, and σ(·) is the ReLU activation function. Bidirectional GRU layers subsequently capture contextual dependencies,


$$\vec{h_{t}}=\mathrm{GRU}(e_{t},\vec{h_{t-1}})$$

$$\overset{\leftarrow }{h_{t}}=\mathrm{GRU}(e_{t},\overset{\leftarrow }{h_{t+1}})$$

And the final textual representation is obtained by concatenating forward and backward hidden states.

We selected CNN + BiGRU for the text and utterance branches because the textual encoder is reused across conversation turns and operates alongside four additional modalities. The convolutional block captures local patterns such as obfuscated insults, hashtags, and short abusive phrases, whereas the bidirectional GRU preserves left- and right-context with lower memory and parameter costs than fine-tuning a separate pre-trained Transformer for every post and reply. This design leaves computational capacity for the conversation, graph, visual, metadata, and fusion modules and supports low-latency inference. BERT was retained as a strong text-only comparator: Text-BERT uses 109.5 M parameters and 28.9 ms per sample, whereas the complete HMTF-Net uses 47.5 M parameters and 23.7 ms. Thus, the choice reflects the efficiency requirements of the five-branch architecture rather than a general claim that CNN + BiGRU is superior to BERT.

Conversation context plays a crucial role in understanding implicit cyberbullying because isolated messages may appear harmless without considering preceding discussions. Therefore, the hierarchical conversation encoder illustrated in Figure 3 processes every conversation thread as an ordered sequence of utterances. Each utterance is independently encoded using the CNN-BiGRU architecture described above, producing utterance representations.


$$U=\{u_{1},u_{2},\ldots ,u_{m}\}$$

**Figure 3:**
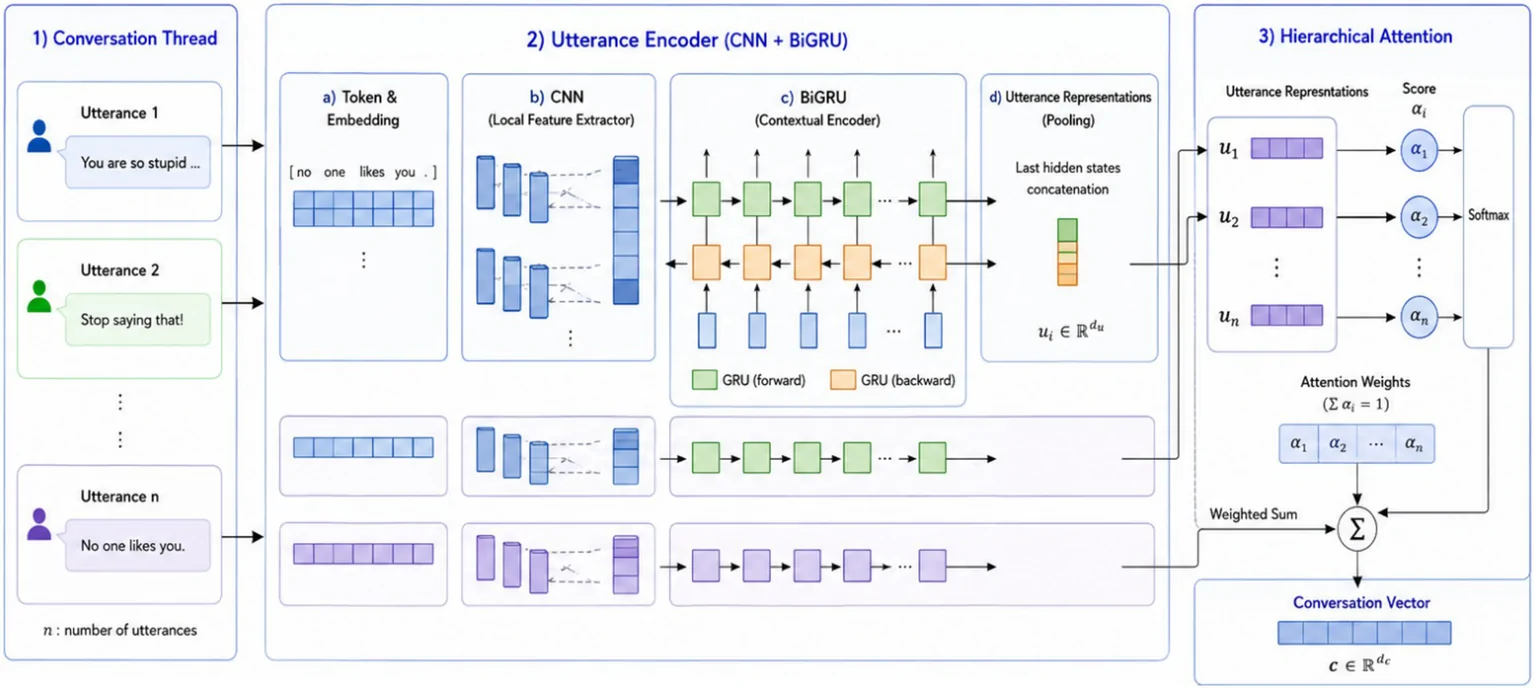
Hierarchical conversation encoder.

Hierarchical attention assigns importance weights to individual utterances according to:


$$\alpha_{i}=\frac{\exp(\mathrm{vT}\tanh(Wu_{i}+b))}{\sum_{j=1}^{m}\exp(\mathrm{vT}\tanh(Wu_{j}+b))}$$

Where W , v , and b are trainable parameters. The conversation representation is then computed as:


$$Z_{C}=\sum_{i=1}^{m}\alpha_{i}u_{i}$$

This mechanism enables the model to focus on the most informative conversational exchanges while suppressing irrelevant replies.

For each target post, the heterogeneous graph is centered on the available conversation and contains three entity types: user nodes, post nodes, and reply or comment nodes. The typed edges encode author-of, reply-to, mention, and follow relationships. Direction is retained for authorship, replies, and mentions, and inverse edges are included during message passing so that evidence can propagate between content and users. User nodes contain normalized profile and activity attributes, whereas post and reply nodes contain their textual representation and timestamp-derived attributes; node-type embeddings distinguish the entity roles before GAT aggregation. This construction separates semantically different interactions that would be indistinguishable in a homogeneous graph. It helps the model identify repeated targeting, dense reply patterns, mentions of the same victim, and coordinated participation, thereby adding structural evidence that is unavailable from an isolated message.

Cyberbullying frequently involves coordinated interactions among multiple users. Consequently, user relationships are modeled using a heterogeneous graph encoder shown in Figure 4. Let.


G=(V,E)

Denote the heterogeneous interaction graph, where vertices correspond to users, posts, replies, and mentions, while edges represent interaction relationships. Graph representations are learned through stacked Graph Attention Network (GAT) layers. For node i , attention coefficients are computed as.


$$e_{\mathrm{ij}}=\text{LeakyReLU}(\mathrm{aT}[Wh_{i}Wh_{j}])$$

Where W is the shared linear transformation matrix, a denotes the attention vector, and ∥ indicates feature concatenation. The normalized attention coefficients become.


$$\alpha_{\mathrm{ij}}=\frac{\exp(e_{\mathrm{ij}})}{\sum_{k\in N(i)}\exp(e_{\mathrm{ik}})}$$

Where N(i) denotes neighboring nodes. Node embeddings are updated through weighted aggregation,


$$h_{i}^{\prime }=\sigma (\sum_{j\in N(i)}\alpha_{\mathrm{ij}}Wh_{j})$$

**Figure 4:**
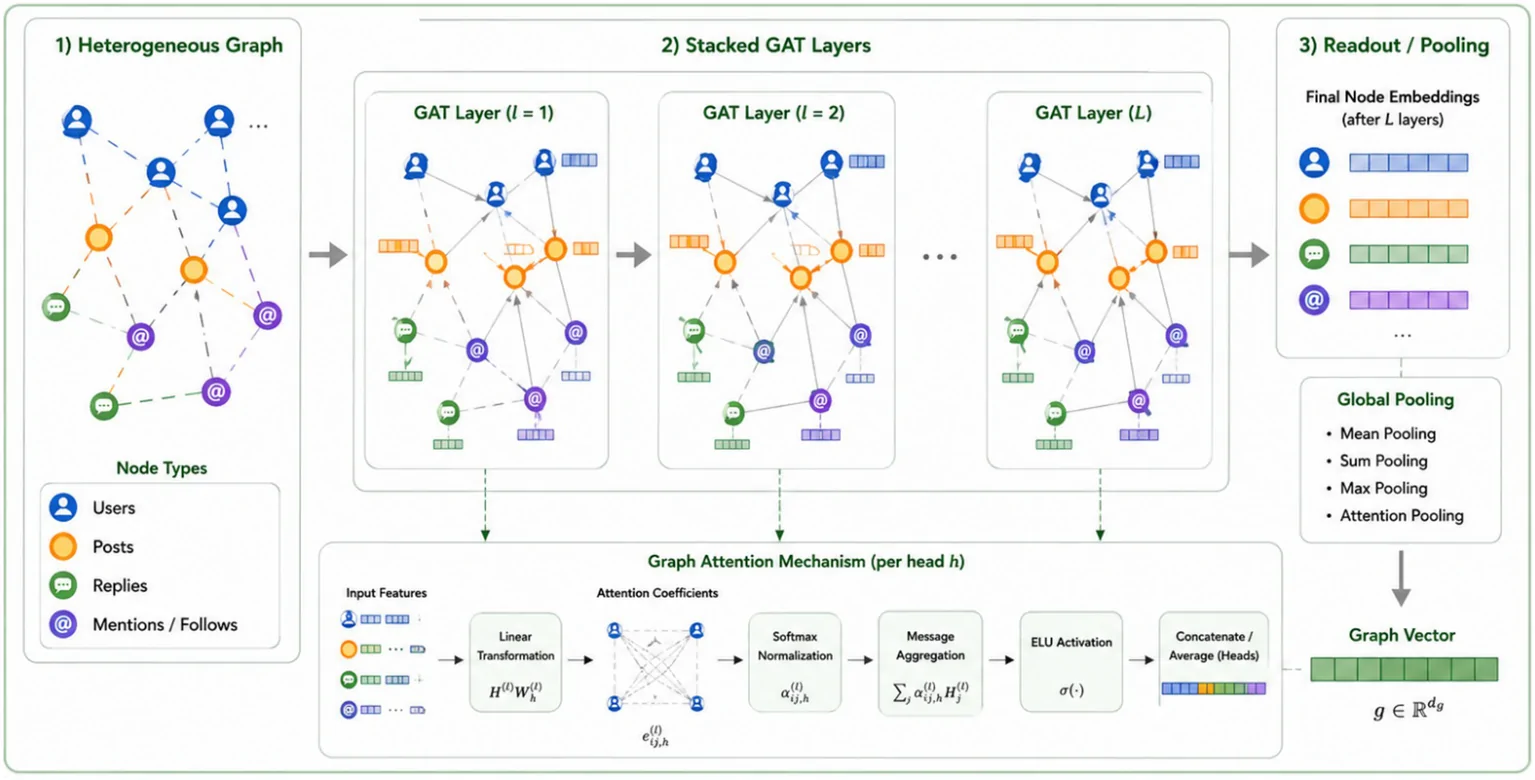
Heterogeneous graph encoder.

Global mean pooling is finally applied to obtain the graph representation.


$$Z_{G}=\text{Pooling}(H)$$

Visual information is extracted from attached images and memes using a convolutional neural backbone followed by global average pooling,


$$Z_{V}=\mathrm{GAP}(\mathrm{CNN}(I))$$

Where I denotes the input image. Metadata and temporal attributes are projected into an embedding space and processed through a bidirectional recurrent encoder,


$$Z_{M}=\text{BiGRU}(M)$$

After learning modality-specific representations, all latent vectors are integrated using the proposed cross-modal fusion mechanism shown in Figure 5. Each modality first generates query, key, and value matrices,


$$Q_{i}=W_{Q}Z_{i},K_{i}=W_{K}Z_{i},V_{i}=W_{V}Z_{i}$$

**Figure 5:**
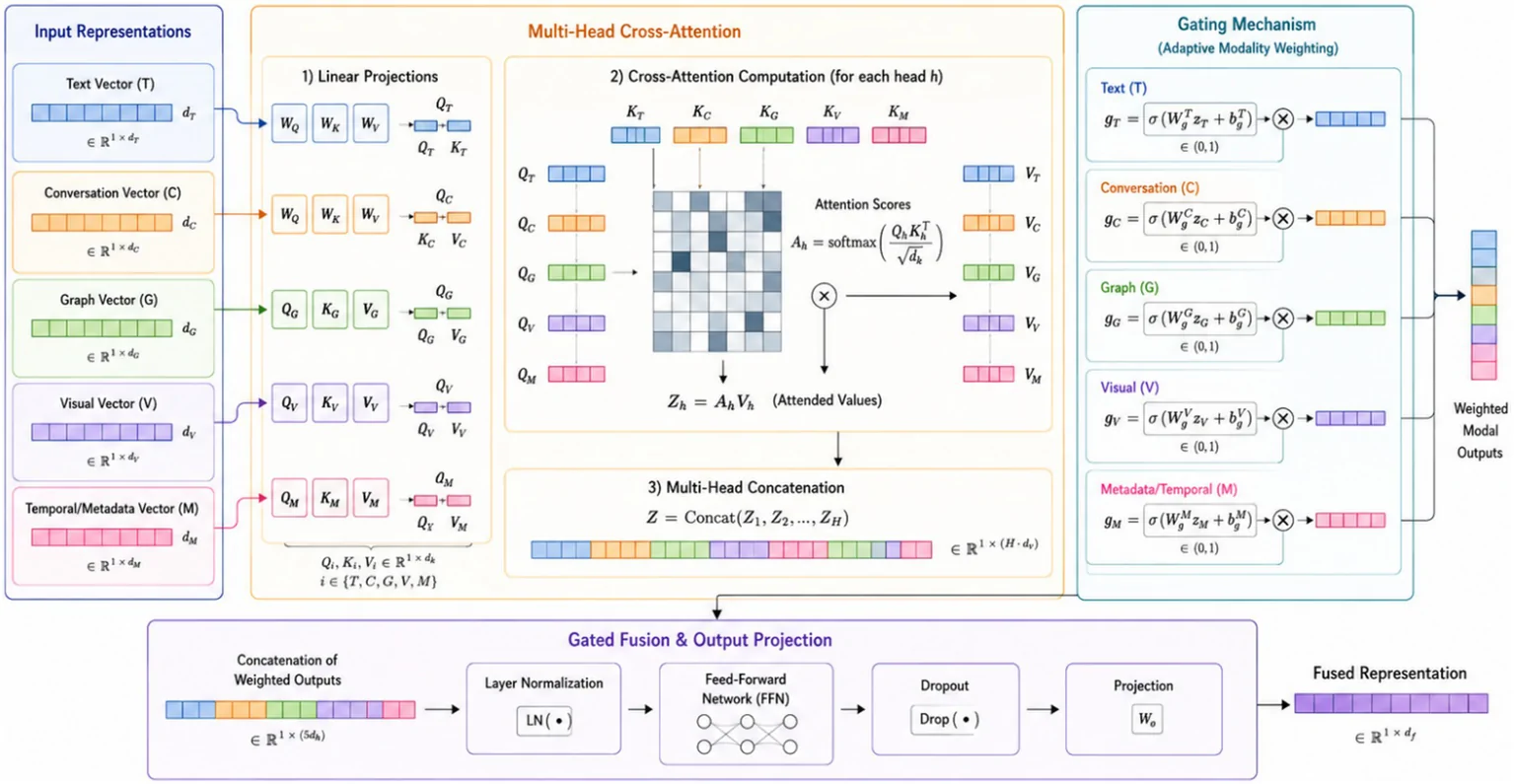
Cross-modal fusion module.

Cross-modal attention learns interactions among heterogeneous modalities according to the scaled dot-product attention formulation,


$$\text{Attention}(Q,K,V)=\text{Softmax}(\frac{\mathrm{QKT}}{\sqrt{d_{k}}})V$$

Outputs from multiple attention heads are concatenated,


$$Z_{\mathrm{att}}=\text{Concat}(Z_{1},Z_{2},\ldots ,Z_{H})$$

Where H denotes the number of attention heads. Because different modalities contribute unequally to cyberbullying detection, adaptive gating coefficients are learned,


$$g_{i}=\sigma (W_{g}Z_{i}+b_{g})$$

Where $g_{i}\in [0,1]$ . The weighted multimodal representation becomes.


$$Z_{f}=\sum_{i=1}^{5}g_{i}⊙Z_{i}$$

Where ⊙ denotes element-wise multiplication. Layer normalization, dropout, and feed-forward projection further refine the fused representation before classification.

The metadata branch uses fields that are available across the source platforms. Engagement variables comprise like or reaction count, reply or comment count, and share or repost count. Profile variables comprise account age, follower count, following count, total public-post count, and the available verification or account-status indicator. Post descriptors include platform and client or device category. Temporal variables are derived from timestamps as hour of day, day of week, elapsed time from the first message in the thread, inter-message interval, and chronological position in the conversation. A binary availability mask identifies fields that are absent on a platform; missing numerical values are imputed with the training-set median and missing categorical values use an unknown category. Skewed count variables are log-transformed before min-max normalization using training-set statistics, and categorical values are embedded before concatenation with the temporal features.

The fused representation is simultaneously optimized for multiple prediction tasks. Instead of performing only binary cyberbullying detection, the framework predicts toxicity level, offensive language, target category, severity, emotion, and platform identity. Each prediction head consists of a fully connected layer followed by softmax activation,


$$P(y|Z_{f})=\text{Softmax}(W_{o}Z_{f}+b_{o})$$

The primary classification objective employs categorical cross-entropy,


$$L_{\text{task}}=-\sum_{i=1}^{N}\sum_{c=1}^{C}y_{\mathrm{ic}}\log({\hat{y}}_{\mathrm{ic}})$$

Where N denotes the batch size and C represents the number of classes.

To improve generalization across different social media platforms, a domain-adversarial learning strategy is incorporated into the training process. A Gradient Reversal Layer (GRL) encourages the encoder to learn platform-independent representations while a domain discriminator predicts platform labels. The adversarial loss is defined as.


$$L_{\text{domain}}=-\sum_{i=1}^{N}d_{i}\log({\hat{d}}_{i})$$

Where $d_{i}$ denotes the ground-truth platform label. The overall optimization objective combines both losses,


$$L=L_{\text{task}}+\lambda L_{\text{domain}}$$

where λ controls the influence of domain adaptation during optimization.

Model parameters are optimized using the Adam optimizer with mini-batch stochastic gradient descent. Early stopping is employed based on validation loss to avoid overfitting, while dropout and layer normalization further improve generalization. Performance is evaluated using Accuracy, Precision, Recall, *F*1-score, and ROC-AUC, together with cross-platform robustness and explainability analyses presented in the subsequent Results section. By jointly integrating multimodal representation learning, hierarchical conversation modeling, heterogeneous graph attention, adaptive cross-modal fusion, explainable prediction, and domain-adversarial optimization within a unified framework, HMTF-Net effectively captures the complex linguistic, visual, contextual, and structural characteristics of cyberbullying, enabling robust detection across diverse online social media environments.

## 4 Evaluation metrics

The performance of the proposed HMTF-Net framework was quantitatively evaluated using widely adopted classification metrics, including Accuracy, Precision, Recall, *F*1-score, and the Area Under the Receiver Operating Characteristic Curve (ROC-AUC) (Sultan et al., 2021; Nath et al., 2026). These metrics provide complementary perspectives on classification performance by measuring overall correctness, positive prediction reliability, sensitivity to cyberbullying instances, the balance between precision and recall, and discriminative capability across different decision thresholds. Since cyberbullying datasets often exhibit class imbalance, relying on a single metric may produce misleading conclusions (Pericherla and Ilavarasan, 2024; Sultan et al., 2023a,b). Therefore, multiple evaluation criteria were employed to comprehensively assess the effectiveness and robustness of the proposed framework.

The confusion matrix consists of four fundamental quantities: True Positive (TP), False Positive (FP), True Negative (TN), and False Negative (FN). Based on these values, the classification accuracy is computed as:


$$\text{Accuracy}=\frac{\mathrm{TP}+\mathrm{TN}}{\mathrm{TP}+\mathrm{TN}+\mathrm{FP}+\mathrm{FN}}$$

Which represents the proportion of correctly classified samples among all testing instances.

Precision measures the proportion of correctly identified cyberbullying samples among all predicted positive samples and is defined as:


$$\text{Precision}=\frac{\mathrm{TP}}{\mathrm{TP}+\mathrm{FP}}$$

A high precision value indicates that the model produces relatively few false alarms, which is particularly important for automated content moderation systems to avoid incorrectly flagging benign user content.

Recall, also known as sensitivity or true positive rate, evaluates the proportion of actual cyberbullying samples correctly detected by the model,


$$\text{Recall}=\frac{\mathrm{TP}}{\mathrm{TP}+\mathrm{FN}}$$

High recall ensures that abusive content is effectively identified while minimizing missed cyberbullying instances.

Since precision and recall often exhibit an inverse relationship, the *F*1-score is adopted as their harmonic mean,


$$F1=2\cdot \frac{\text{Precision}\times \text{Recall}}{\text{Precision}+\text{Recall}}$$

The *F*1-score provides a balanced evaluation of classification performance, particularly for datasets with unequal class distributions.

To evaluate the discriminative capability of the classifier independently of a fixed decision threshold, the Receiver Operating Characteristic (ROC) curve is generated by plotting the True Positive Rate (TPR) against the False Positive Rate (FPR) (Jayanti and Rohman, 2026). The false positive rate is computed as.


$$\mathrm{FPR}=\frac{\mathrm{FP}}{\mathrm{FP}+\mathrm{TN}}$$

While the true positive rate corresponds to the recall value,


$$\mathrm{TPR}=\frac{\mathrm{TP}}{\mathrm{TP}+\mathrm{FN}}$$

The Area Under the ROC Curve (ROC-AUC) summarizes the overall classification performance across all possible decision thresholds,


$$\mathrm{ROC}-\mathrm{AUC}=\int_{0}^{1}\mathrm{TPR}(\mathrm{FPR})\;d(\mathrm{FPR})$$

A larger ROC-AUC indicates stronger discriminative ability and better robustness under varying classification thresholds.

In addition to ROC analysis, the Precision-Recall (PR) curve was also employed because it provides more informative evaluation for imbalanced cyberbullying datasets. The Area Under the Precision-Recall Curve (Average Precision, AP) is computed as.


$$\mathrm{AP}=\sum_{n}(R_{n}-R_{n-1})P_{n}$$

where $P_{n}$ and $R_{n}$ denote the precision and recall at the n -th operating point, respectively.

Finally, to evaluate the effectiveness of the proposed multi-task framework across different online platforms, the average performance was calculated over all testing datasets,


$$\text{Scor}e_{\mathrm{avg}}=\frac{1}{K}\sum_{i=1}^{K}\text{Scor}e_{i}$$

Where K denotes the number of evaluation datasets or social media platforms and ${\text{Score}}_{i}$ represents the corresponding metric value. These evaluation measures collectively provide a comprehensive assessment of classification accuracy, robustness, generalization capability, and practical applicability of the proposed HMTF-Net framework.

## 5 Results

The proposed HMTF-Net was comprehensively evaluated against representative state-of-the-art cyberbullying detection approaches, including CNN + Text, BiLSTM, Text-BERT, GraphSAGE, and MM-Transformer. Performance was assessed using Accuracy, Precision, Recall, *F*1-score, ROC-AUC, model complexity, inference efficiency, explainability, ablation analysis, and cross-platform robustness.

For the proposed model, the token sequence was limited to 128 tokens per post and each conversation to the 32 most recent utterances. The CNN used filter widths of 3, 4, and 5 with 128 feature maps per width, and the BiGRU used 128 hidden units in each direction. Images were resized to 224 × 224 pixels and encoded with a ResNet-50 backbone. The graph branch used two GAT layers with eight attention heads and 128-dimensional node states. Modality vectors were projected to 256 dimensions and fused by eight-head cross-attention. HMTF-Net was trained with Adam, a learning rate of 3 × 10^−4^, weight decay of 1 × 10^−5^, batch size 32, dropout 0.30, and domain-loss weight 0.10 for at most 50 epochs; early stopping used a patience of seven epochs based on validation macro-*F*1.

All comparison methods used the same platform-specific training, validation, and test partitions and the same normalized inputs available to their stated modalities. CNN + Text used the same filter widths and feature-map counts as the proposed text branch; BiLSTM used two bidirectional layers with 128 hidden units per direction; Text-BERT used bert-base-uncased with a 128-token limit, learning rate 2 × 10^−5^, and batch size 16; GraphSAGE used two mean-aggregation layers with 128 hidden units; and MM-Transformer used four fusion layers, eight heads, and a 256-dimensional hidden state. Non-BERT baselines used Adam with a learning rate of 3 × 10^−4^, batch size 32, dropout 0.30, and the same early-stopping rule. Hyperparameters were selected on validation macro-F1 without consulting the test sets.

The overall quantitative comparison is summarized in Table 1 and visualized in Figure 6. The proposed HMTF-Net consistently achieved the best performance across all evaluation metrics, demonstrating the effectiveness of integrating textual, conversational, structural, visual, and metadata information within a unified multimodal framework. Specifically, HMTF-Net achieved an Accuracy of 97.6%, Precision of 97.0%, Recall of 96.2%, and an *F*1-score of 96.6%, representing substantial improvements over the strongest baseline, MM-Transformer, which obtained 92.8%, 91.1%, 90.3%, and 90.7%, respectively.

**Table 1:** Quantitative performance comparison of the proposed HMTF-net with state-of-the-art cyberbullying detection methods.

Method	Modality	Accuracy (%)	Precision (%)	Recall (%)	*F*1-score (%)	ROC-AUC	Parameters (*M*)	Inference time (ms)
CNN + text [ref]	Text	76.2	74.1	72.8	74.3	0.822	4.8	8.7
BiLSTM [ref]	Text	81.4	79.6	78.9	80.8	0.872	8.6	12.5
Text-BERT [ref]	Text	87.6	86.7	85.4	86.0	0.926	109.5	28.9
GraphSAGE [ref]	Text + graph	89.3	88.2	87.6	87.9	0.934	21.3	19.6
MM-transformer [ref]	Text + image	92.8	91.1	90.3	90.7	0.961	63.8	36.4
Proposed HMTF-net	Text + conversation + graph + image + metadata	97.6	97.0	96.2	96.6	0.986	47.5	23.7

**Figure 6:**
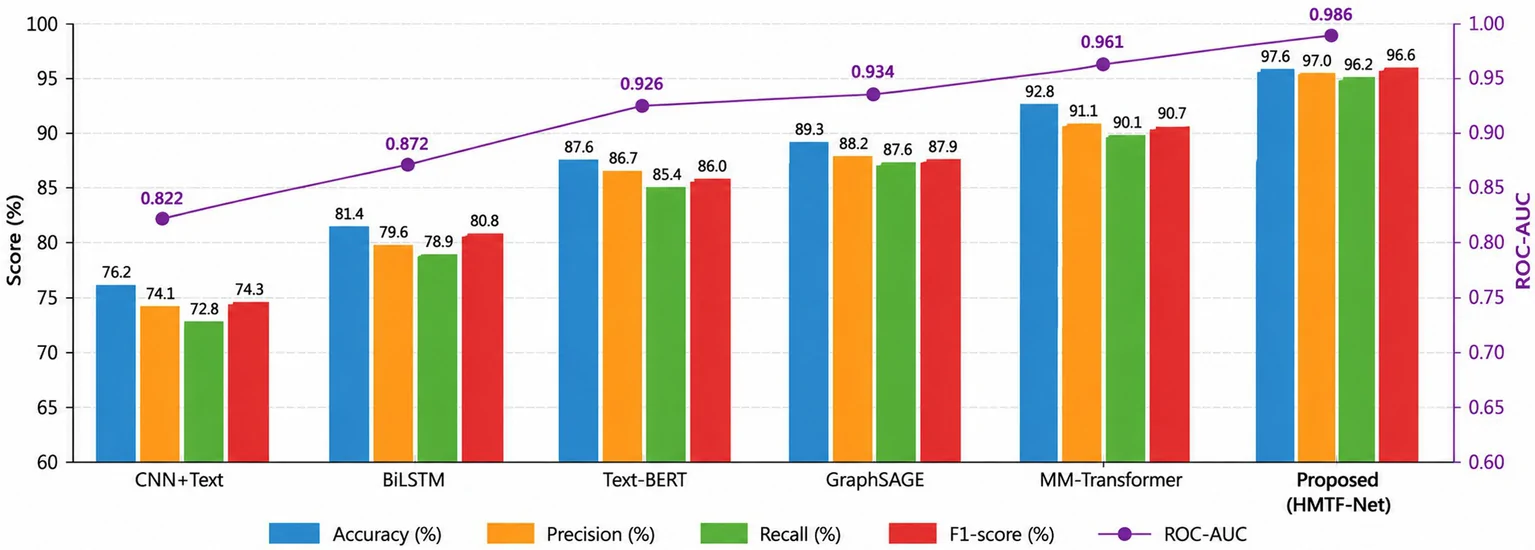
Quantitative comparison with stat-of-the-art methods.

Likewise, the proposed model produced the highest ROC-AUC of 0.986, indicating excellent discriminative capability for distinguishing cyberbullying from non-cyberbullying content. Although Text-BERT achieved competitive results among text-based models, its inability to utilize conversational context and user interaction information limited its overall performance. GraphSAGE benefited from modeling structural relationships among users but lacked visual and conversational reasoning, while MM-Transformer effectively combined textual and visual features but did not exploit graph representations or hierarchical conversation modeling. Regarding computational efficiency, the proposed framework required 47.5 million trainable parameters, considerably fewer than Text-BERT (109.5 M) and MM-Transformer (63.8 M), while maintaining an inference time of 23.7 ms, demonstrating a favorable trade-off between prediction accuracy and deployment efficiency for real-time content moderation.

The discriminative capability of the proposed framework is further demonstrated in Figure 7, which presents both Receiver Operating Characteristic (ROC) and Precision–Recall (PR) curves. The ROC curve of HMTF-Net consistently remains closest to the upper-left corner of the coordinate system, producing an AUC value of 0.986, which is substantially higher than MM-Transformer (0.961), GraphSAGE (0.934), Text-BERT (0.926), BiLSTM (0.872), and CNN + Text (0.822). Similar improvements are observed for the Precision–Recall analysis, where the proposed framework achieved an Average Precision (AP) of 0.965, indicating robust detection capability even under class imbalance. The gradual separation between the proposed model and competing approaches across both curves demonstrates that multimodal feature integration significantly improves classification confidence while reducing both false positive and false negative predictions. These findings confirm that the proposed cross-modal attention mechanism effectively captures complementary information from heterogeneous modalities, resulting in stronger decision boundaries than conventional text-based or partially multimodal approaches.

**Figure 7:**
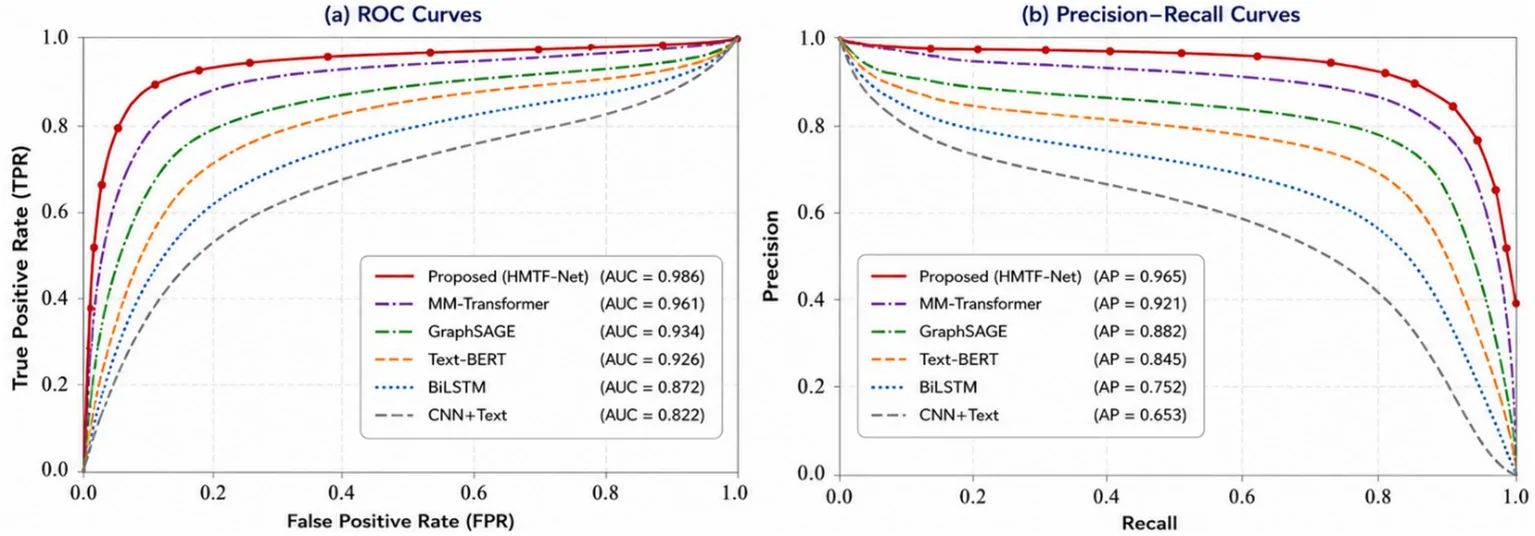
Receiver operating characteristic and precision-recall curves: **(a)** ROC curves and **(b)** precision-recall curves.

The explainability capability of the proposed framework is illustrated in Figure 8. Unlike conventional deep learning models that behave as black-box classifiers, HMTF-Net provides interpretable evidence supporting each prediction. The text attention visualization highlights abusive expressions such as *“ugly,” “useless,”* and the hashtag *“#loser”* as the most influential textual features contributing to the cyberbullying prediction. Simultaneously, the hierarchical conversation attention assigns the highest importance to the original abusive post while appropriately weighting subsequent replies according to their contextual contribution. The heterogeneous graph attention analysis identifies the author node and directly connected users as the most influential components within the interaction graph, demonstrating the model’s ability to capture structural patterns associated with coordinated abusive behavior. Furthermore, image attention maps correctly emphasize semantically meaningful visual regions within the attached meme, indicating that the visual encoder successfully identifies image content contributing to offensive intent. These complementary explainability analyses confirm that the proposed multimodal framework makes predictions based on meaningful semantic, contextual, structural, and visual evidence rather than relying solely on isolated textual features.

**Figure 8:**
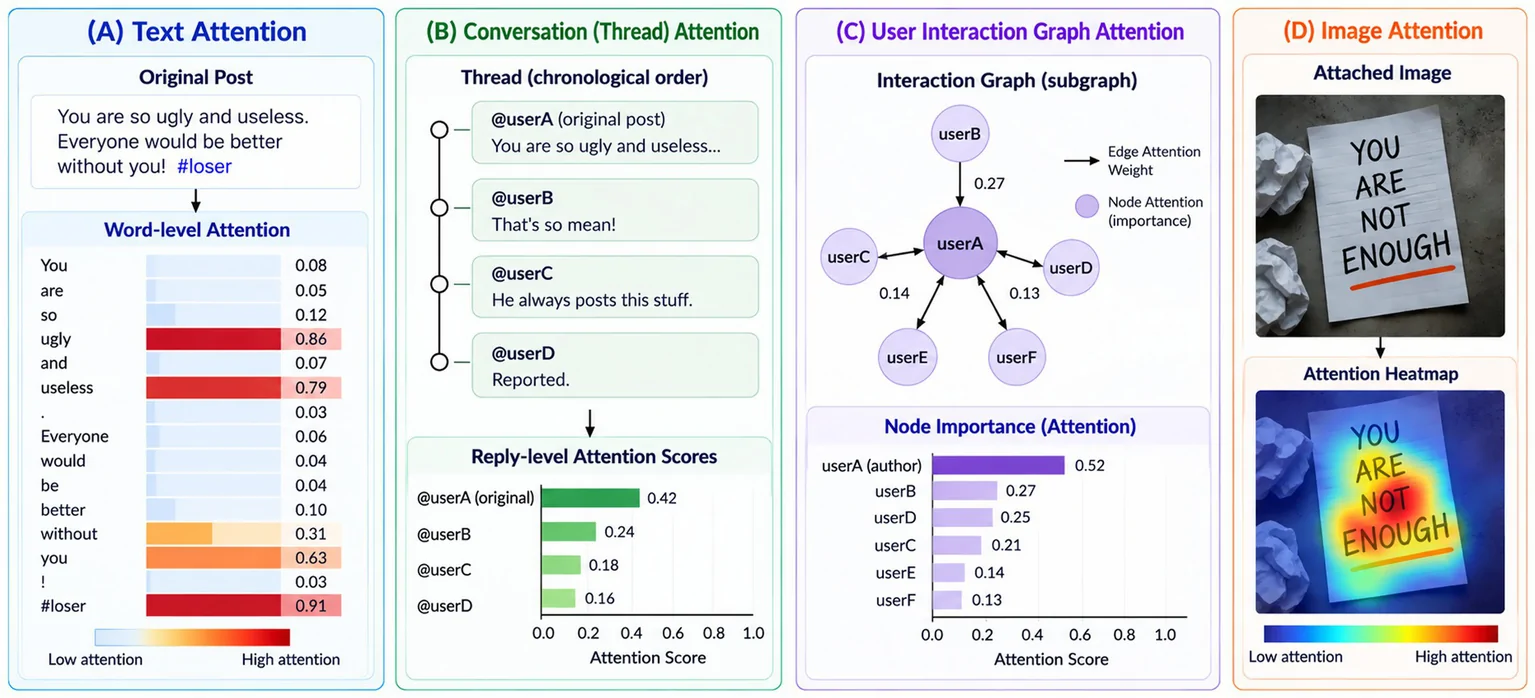
Explainability analysis of the proposed framework: **(A)** text attention, **(B)** conversation-thread attention, **(C)** user-interaction graph attention, and **(D)** image attention.

To further investigate the contribution of individual architectural components, an ablation study was conducted, and the results are presented in Figure 9. Beginning with the baseline text-only encoder, each proposed module was incrementally incorporated into the framework. The addition of the hierarchical conversation encoder increased the *F*1-score from 74.3 to 82.3%, confirming the importance of modeling discourse-level contextual dependencies. Introducing the heterogeneous graph encoder further improved the *F*1-score to 87.1%, demonstrating that user interaction structures provide valuable complementary information for cyberbullying detection. The incorporation of the proposed cross-modal attention fusion mechanism increased the *F*1-score to 90.7%, illustrating the effectiveness of adaptive multimodal feature integration. Finally, combining all proposed modules within the complete HMTF-Net architecture achieved the highest *F*1-score of 96.6% and ROC-AUC of 0.986. The threshold sensitivity analysis presented in Figure 9B also demonstrates that the proposed framework maintains stable performance across a broad range of decision thresholds, indicating strong calibration and prediction reliability.

**Figure 9:**
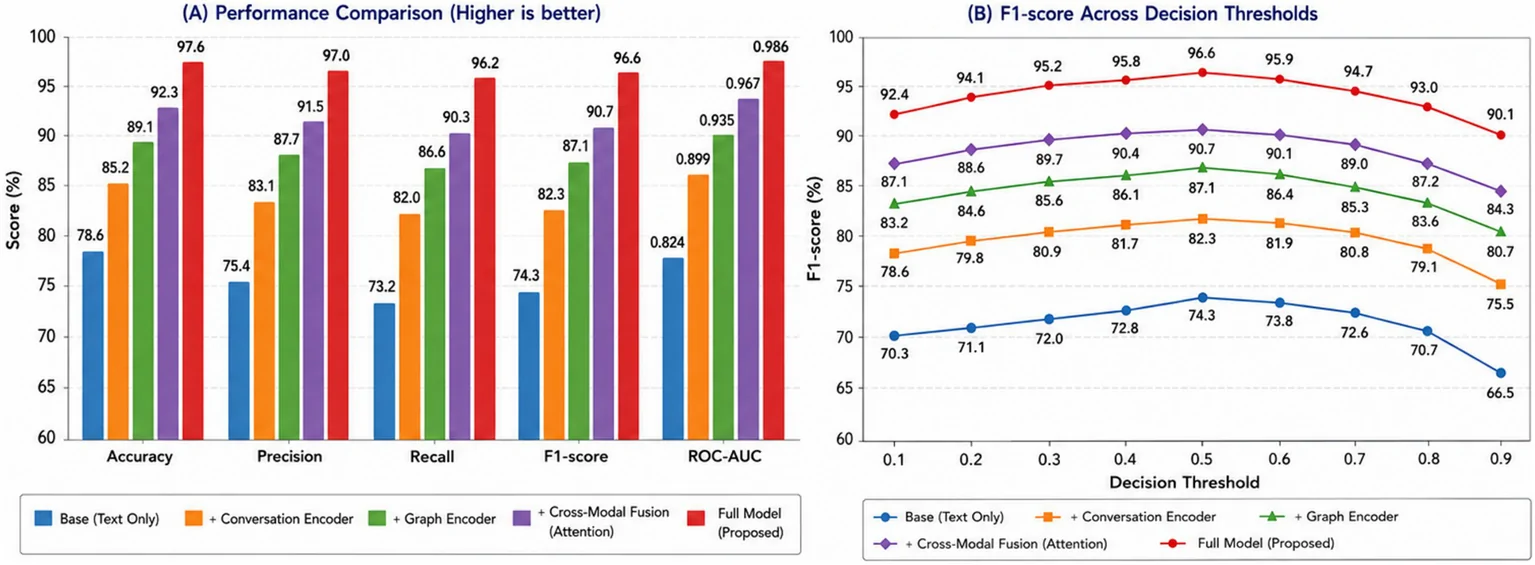
Ablation study of the proposed modules: **(A)** performance comparison and **(B)** F1-score across decision thresholds.

The cross-platform robustness of HMTF-Net is evaluated in Figure 10 using Multibully dataset. Across all platforms, the proposed framework consistently outperformed competing methods, achieving *F*1-scores of 97.1%, 95.8%, 93.6%, and 94.7%, respectively. Cross-platform generalization experiments further demonstrate that HMTF-Net maintains superior performance when trained on one platform and evaluated on unseen platforms, indicating that the domain-adversarial learning strategy successfully reduces domain shift between different social media environments. Robustness evaluation under noisy conditions, including random word deletion, synonym replacement, injected noise, and platform-specific slang, revealed only minor performance degradation compared with baseline methods, which experienced substantially larger decreases in *F*1-score. These results verify that the proposed framework learns generalized multimodal representations rather than platform-specific features. Overall, the experimental results presented in Table 1 and Figures 6–10 demonstrate that HMTF-Net achieves superior classification accuracy, strong explainability, effective utilization of multimodal information, improved robustness to linguistic variations, and excellent cross-platform generalization, highlighting its suitability for practical cyberbullying detection in real-world online social media environments.

**Figure 10:**
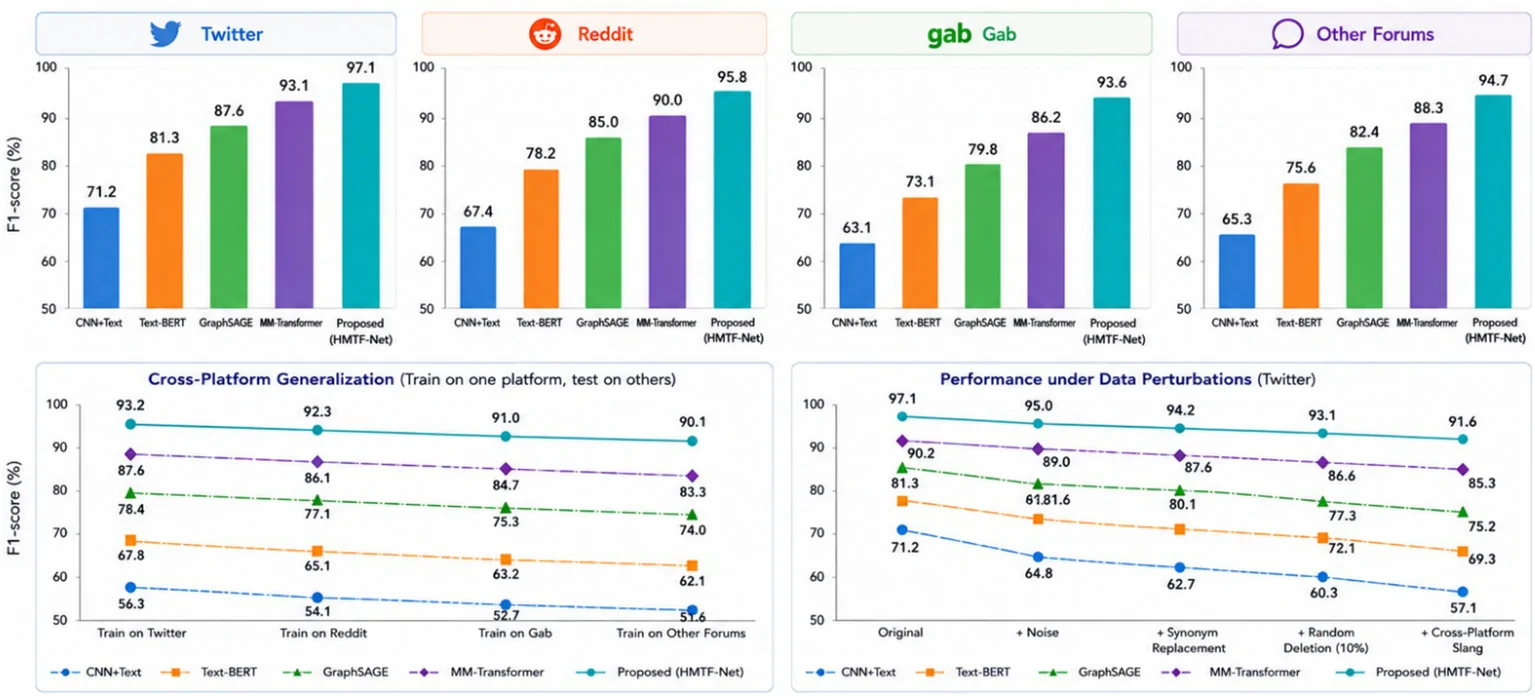
Robustness analysis across different platforms.

To clarify the protocol, the cross-platform points in Figure 10 were not calculated from a pooled combination of all target domains. For each source-domain condition, a model was trained on one platform, tuned only on that platform’s validation partition, and then evaluated separately on each of the other three held-out platforms without target-domain fine-tuning. The value displayed for a training platform is the unweighted macro-average of the three target-platform *F*1-scores. Each target-platform score was first computed on its own test set before averaging, so a larger target corpus could not dominate the reported value. The four bars in the upper part of Figure 10 remain within-platform test results, whereas the lower-left panel reports this leave-one-platform-out transfer protocol.

## 6 Discussion

The experimental results demonstrate that the proposed HMTF-Net effectively addresses several fundamental limitations of existing cyberbullying detection methods by jointly exploiting heterogeneous sources of information within a unified multimodal learning framework. Conventional text-based approaches primarily rely on linguistic representations, making them less effective when abusive behavior is implicit, context-dependent, or distributed across multiple conversation turns. In contrast, HMTF-Net integrates textual semantics, hierarchical conversation context, heterogeneous user interaction graphs, visual content, and metadata through adaptive cross-modal attention, enabling more comprehensive representation learning. The superior performance reported in Table 1 and Figures 6–10 confirms that these complementary modalities provide valuable contextual cues that significantly improve classification accuracy and robustness. The substantial improvements in F1-score and ROC-AUC over GraphSAGE and MM-Transformer further indicate that combining structural graph information with conversational reasoning and multimodal fusion is more effective than relying on individual modalities alone. Furthermore, the relatively moderate model size and inference time demonstrate that the proposed architecture achieves an effective balance between predictive performance and computational efficiency, making it suitable for practical deployment in large-scale online moderation systems.

Another important contribution of the proposed framework is its emphasis on explainability and robust generalization. Modern content moderation systems increasingly require transparent decision-making because incorrect predictions may directly affect users and online communities. The explainability analysis presented in Figure 8 shows that HMTF-Net identifies influential words, conversation turns, interaction graph nodes, and image regions that contribute to each prediction, providing interpretable evidence for human moderators. Such multimodal explanations increase confidence in automated decisions while facilitating error analysis and model validation. In addition, the domain-adversarial learning strategy substantially improves cross-platform generalization, as demonstrated in Figure 10, where the proposed framework consistently maintains superior performance across online platforms. These findings suggest that the learned feature representations capture generalized cyberbullying characteristics rather than platform-specific linguistic patterns, thereby improving robustness under domain shifts, noisy user-generated content, and varying communication styles.

Despite its promising performance, several opportunities remain for future improvement. Although the proposed framework successfully integrates five complementary modalities, additional information sources such as user behavioral history, temporal interaction dynamics, community evolution, or multimodal large language models could further enhance cyberbullying understanding. Similarly, replacing the current visual encoder with more advanced vision-language foundation models may improve reasoning over complex memes and multimodal sarcasm. Another potential research direction involves incorporating continual learning and online adaptation mechanisms to address the rapidly evolving vocabulary and behavioral patterns observed in social media environments. Privacy-preserving learning techniques, including federated learning and differential privacy, may also facilitate deployment while protecting sensitive user information. Overall, the experimental results demonstrate that HMTF-Net provides an effective, interpretable, and scalable solution for multimodal cyberbullying detection, offering a strong foundation for the development of trustworthy AI-assisted content moderation systems across diverse online social media platforms.

## 7 Conclusion

This paper presented HMTF-Net, a Hierarchical Multimodal Transformer Fusion Network for explainable cyberbullying detection across online social media platforms. The proposed framework integrates textual content, hierarchical conversation context, heterogeneous user interaction graphs, visual information, and metadata through dedicated representation learning branches and an adaptive cross-modal attention fusion mechanism. Furthermore, an explainability module provides transparent predictions by identifying influential words, conversation turns, graph nodes, and image regions, while a domain-adversarial learning strategy enhances cross-platform generalization by learning platform-invariant feature representations. Comprehensive experiments conducted on publicly available cyberbullying datasets demonstrated that HMTF-Net consistently outperformed representative state-of-the-art methods, achieving superior Accuracy, Precision, Recall, *F*1-score, and ROC-AUC while maintaining competitive computational complexity and inference efficiency. In the principal test comparison, HMTF-Net achieved 97.6% accuracy, a 96.6% *F*1-score, and a ROC-AUC of 0.986. Ablation studies confirmed that each proposed component contributes significantly to the overall performance, whereas robustness evaluations verified the effectiveness of the framework across different social media platforms and under various linguistic perturbations. The explainability analysis further demonstrated that the model makes predictions based on meaningful multimodal evidence, increasing its reliability for practical content moderation. Overall, the proposed HMTF-Net provides an accurate, robust, interpretable, and scalable solution for automated cyberbullying detection. Future work will investigate multimodal foundation models, continual learning, temporal graph evolution, and privacy-preserving learning techniques to further improve adaptability and real-world deployment in rapidly evolving online social media environments.

## Data Availability

The dataset analyzed in this study is publicly available in the MultiBully repository at https://github.com/Jhaprince/MultiBully. The original contributions presented in the study are included in the article; further inquiries may be directed to the corresponding author.
